# Supplementary material for: Highly Pathogenic Avian Influenza H5N1 Virus Infections in Wild Red Foxes (Vulpes vulpes) Show Neurotropism and Adaptive Virus Mutations
Source: Microbiol Spectr. 2023 Jan 23;11(1):e02867-22. doi: 10.1128/spectrum.02867-22 (PMC9927208; doi:10.1128/spectrum.02867-22)
Supplement: Supplemental file 1 — Supplemental material. Download spectrum.02867-22-s0001.pdf, PDF file, 0.3 MB [file spectrum.02867-22-s0001.pdf]

Table S1: Immunohistochemistry and histopathology.[a]

	**Fox- Dorst**	**Fox-Heemskerk**	**Fox -Oosterbeek**
**Immunohistochemistry (IHC)-viral protein expression**			
**Nasal conchae**	na	Positive, mild staining mainly in olfactory epithelium	N
**Trachea**	N	N	N
**Lung**	N	N	N
**Heart**	na	Positive, mild staining of cardiomyocytes	N
**Liver**	N	N	N
**Kidney**	N	N	N
**Spleen**	N	N	N
**Stomach/Intestine (colon/jejunum)**	N	N	N
**Brain**	Mild to moderate positive staining in cerebrum, cerebellum negative, bulbus olfactorius na	Mild to moderate positive staining in cerebrum and cerebellum, bulbus olfactorius negative	Mild to moderate staining in cerebrum, cerebellum and bulbus olfactorius are negative
**Hematoxylin and eosin (HE)- evaluation histopathologic changes**			
**Nasal conchae**	na	Mild to moderate necropurulent rhinitis with intraluminal eggs and larvae (most likely *Capillaria spp.)*	Mild to moderate necropurulent rhinitis with intraluminal eggs and larvae (most likely *Capillaria spp.)*

**Trachea**	Severe necropurulent tracheitis with intralesional parasite eggs (most likely Capillaria spp)	Mild purulent (suppurative) tracheitis	nsc
**Lung**	Severe purulent broncho-interstitial pneumonia with lung larvae *(most likely Angiostrongylus vasorum)*	Moderate subacute broncho-interstitial pneumonia with hemorrhage and fibrin	Severe bronchointerstitial pneumonia with intralesional larvae (most likely *Angiostrongylus vasorum*) and multinucleated giant cells
**Heart**	na	Lymphoplasmacytic myocarditis with mild myocardial degeneration and necrosis	nsc
**Liver**	nsc	nsc	nsc
**Kidney**	nsc	nsc	Chronic moderate lymphoplasmacytic interstitial nephritis
**Spleen**	nsc	nsc	nsc
**Stomach/Intestine (colon/jejunum)**	nsc	nsc	nsc
**Brain**	Moderate non-suppurative encephalitis	Moderate non-suppurative encephalitis	Moderate non-suppurative encephalitis

[a] N: negative, na: not analyzed, nsc: no significant changes

PB2

Figure S1

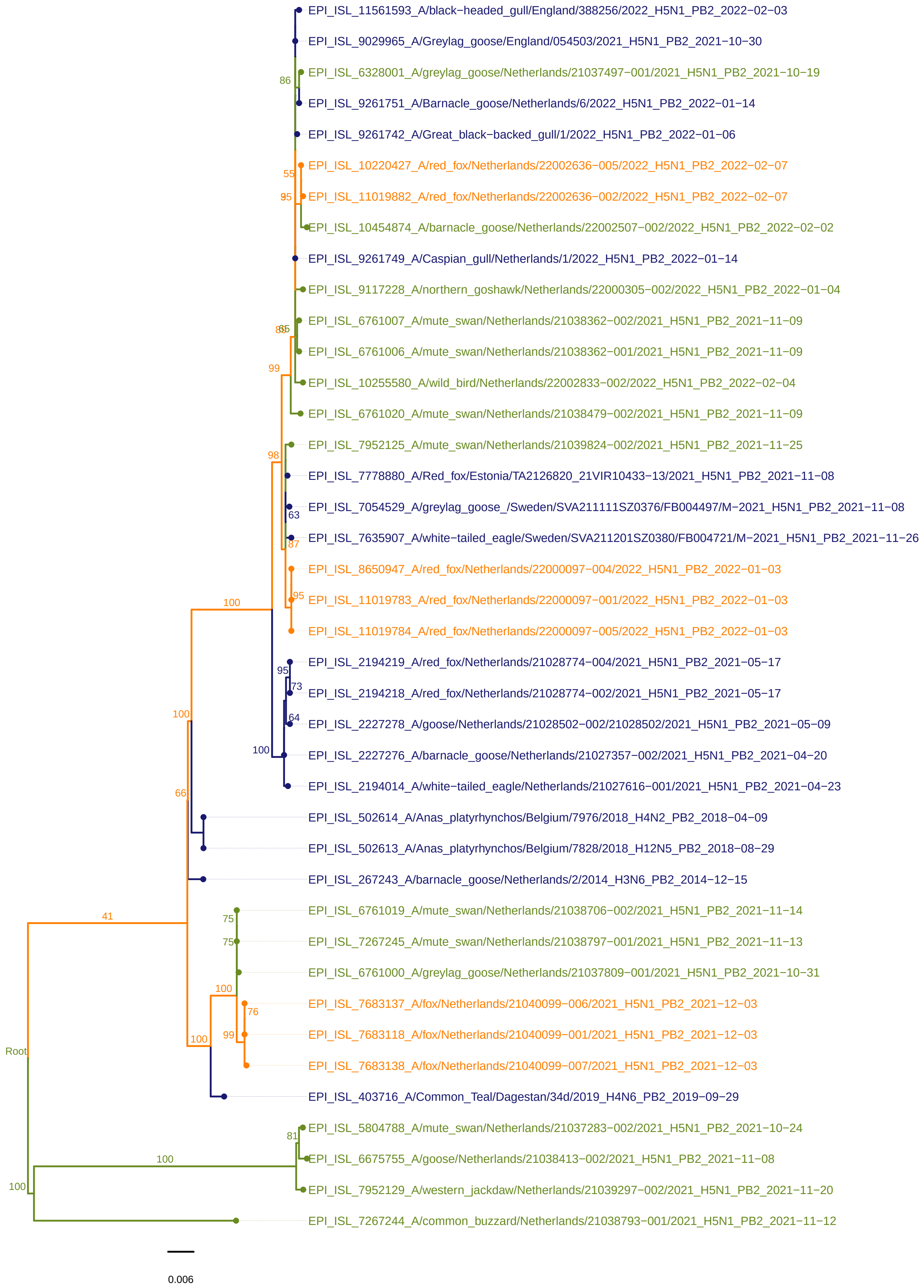

PB1

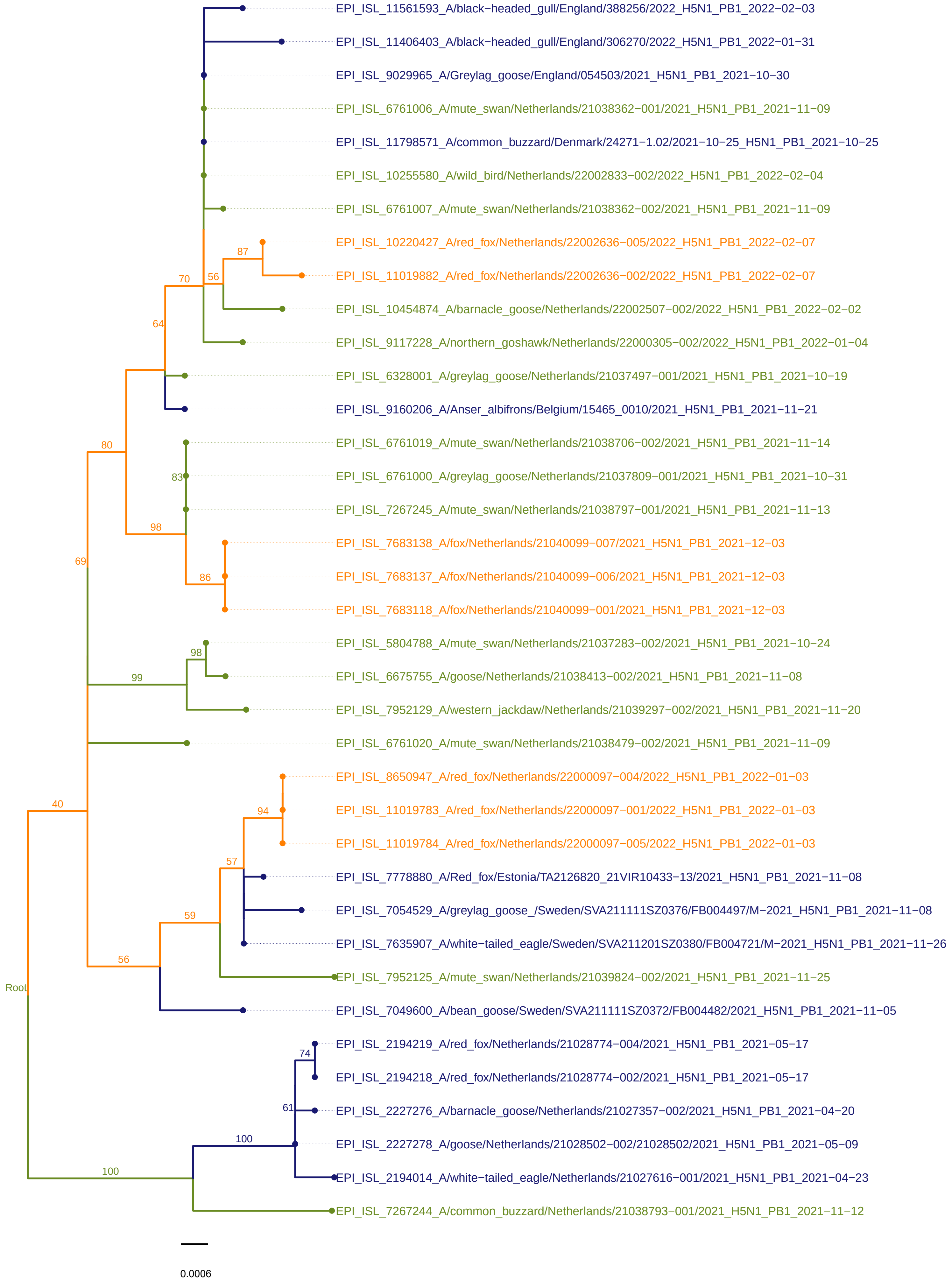
EPI_ISL_11561593_A/black−headed_gull/England/388256/2022_H5N1_PB1_2022−02−03
EPI_ISL_11406403_A/black−headed_gull/England/306270/2022_H5N1_PB1_2022−01−31
EPI_ISL_9029965_A/Greylag_goose/England/054503/2021_H5N1_PB1_2021−10−30
EPI_ISL_6761006_A/mute_swan/Netherlands/21038362−001/2021_H5N1_PB1_2021−11−09
EPI_ISL_11798571_A/common_buzzard/Denmark/24271−1.02/2021−10−25_H5N1_PB1_2021−10−25
EPI_ISL_10255580_A/wild_bird/Netherlands/22002833−002/2022_H5N1_PB1_2022−02−04
EPI_ISL_6761007_A/mute_swan/Netherlands/21038362−002/2021_H5N1_PB1_2021−11−09
EPI_ISL_10220427_A/red_fox/Netherlands/22002636−005/2022_H5N1_PB1_2022−02−07
EPI_ISL_11019882_A/red_fox/Netherlands/22002636−002/2022_H5N1_PB1_2022−02−07
EPI_ISL_10454874_A/barnacle_goose/Netherlands/22002507−002/2022_H5N1_PB1_2022−02−02
EPI_ISL_9117228_A/northern_goshawk/Netherlands/22000305−002/2022_H5N1_PB1_2022−01−04
EPI_ISL_6328001_A/greylag_goose/Netherlands/21037497−001/2021_H5N1_PB1_2021−10−19
EPI_ISL_9160206_A/Anser_albifrons/Belgium/15465_0010/2021_H5N1_PB1_2021−11−21
EPI_ISL_6761019_A/mute_swan/Netherlands/21038706−002/2021_H5N1_PB1_2021−11−14
EPI_ISL_6761000_A/greylag_goose/Netherlands/21037809−001/2021_H5N1_PB1_2021−10−31
EPI_ISL_7267245_A/mute_swan/Netherlands/21038797−001/2021_H5N1_PB1_2021−11−13
EPI_ISL_7683138_A/fox/Netherlands/21040099−007/2021_H5N1_PB1_2021−12−03
EPI_ISL_7683137_A/fox/Netherlands/21040099−006/2021_H5N1_PB1_2021−12−03
EPI_ISL_7683118_A/fox/Netherlands/21040099−001/2021_H5N1_PB1_2021−12−03
EPI_ISL_5804788_A/mute_swan/Netherlands/21037283−002/2021_H5N1_PB1_2021−10−24
EPI_ISL_6675755_A/goose/Netherlands/21038413−002/2021_H5N1_PB1_2021−11−08
EPI_ISL_7952129_A/western_jackdaw/Netherlands/21039297−002/2021_H5N1_PB1_2021−11−20
EPI_ISL_6761020_A/mute_swan/Netherlands/21038479−002/2021_H5N1_PB1_2021−11−09
EPI_ISL_8650947_A/red_fox/Netherlands/22000097−004/2022_H5N1_PB1_2022−01−03
EPI_ISL_11019783_A/red_fox/Netherlands/22000097−001/2022_H5N1_PB1_2022−01−03
EPI_ISL_11019784_A/red_fox/Netherlands/22000097−005/2022_H5N1_PB1_2022−01−03
EPI_ISL_7778880_A/Red_fox/Estonia/TA2126820_21VIR10433−13/2021_H5N1_PB1_2021−11−08
EPI_ISL_7054529_A/greylag_goose_/Sweden/SVA211111SZ0376/FB004497/M−2021_H5N1_PB1_2021−11−08
EPI_ISL_7635907_A/white−tailed_eagle/Sweden/SVA211201SZ0380/FB004721/M−2021_H5N1_PB1_2021−11−26
EPI_ISL_7952125_A/mute_swan/Netherlands/21039824−002/2021_H5N1_PB1_2021−11−25
EPI_ISL_7049600_A/bean_goose/Sweden/SVA211111SZ0372/FB004482/2021_H5N1_PB1_2021−11−05
EPI_ISL_2194219_A/red_fox/Netherlands/21028774−004/2021_H5N1_PB1_2021−05−17
EPI_ISL_2194218_A/red_fox/Netherlands/21028774−002/2021_H5N1_PB1_2021−05−17
EPI_ISL_2227276_A/barnacle_goose/Netherlands/21027357−002/2021_H5N1_PB1_2021−04−20
EPI_ISL_2227278_A/goose/Netherlands/21028502−002/21028502/2021_H5N1_PB1_2021−05−09
EPI_ISL_2194014_A/white−tailed_eagle/Netherlands/21027616−001/2021_H5N1_PB1_2021−04−23
EPI_ISL_7267244_A/common_buzzard/Netherlands/21038793−001/2021_H5N1_PB1_2021−11−12
87
70
56
64
80
83
98
86
69
98
99
40
94
57
59
56
Root
74
61
100
100
0.0006

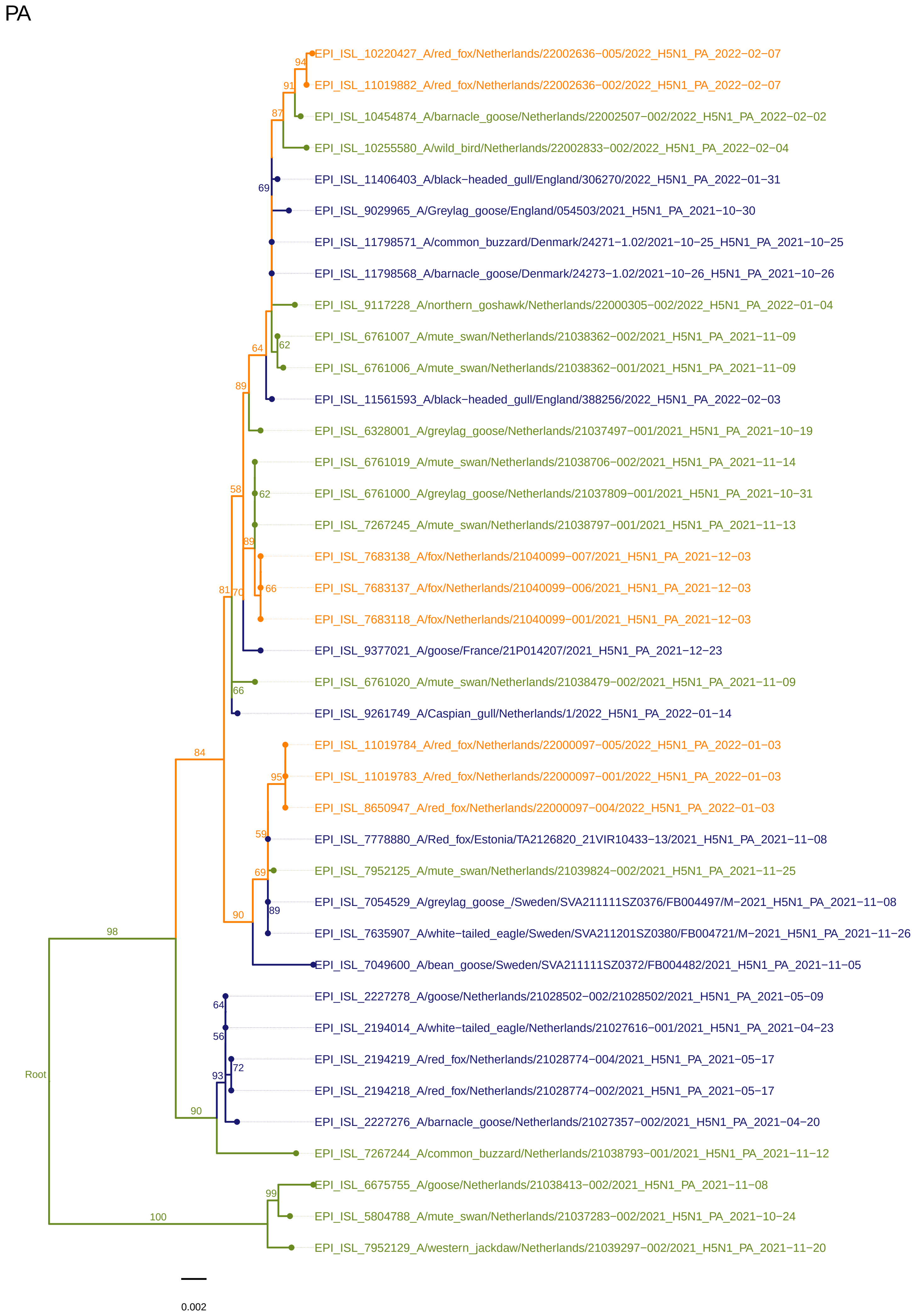
PA
EPI_ISL_10220427_A/red_fox/Netherlands/22002636−005/2022_H5N1_PA_2022−02−07
EPI_ISL_11019882_A/red_fox/Netherlands/22002636−002/2022_H5N1_PA_2022−02−07
EPI_ISL_10454874_A/barnacle_goose/Netherlands/22002507−002/2022_H5N1_PA_2022−02−02
EPI_ISL_10255580_A/wild_bird/Netherlands/22002833−002/2022_H5N1_PA_2022−02−04
EPI_ISL_11406403_A/black−headed_gull/England/306270/2022_H5N1_PA_2022−01−31
EPI_ISL_9029965_A/Greylag_goose/England/054503/2021_H5N1_PA_2021−10−30
EPI_ISL_11798571_A/common_buzzard/Denmark/24271−1.02/2021−10−25_H5N1_PA_2021−10−25
EPI_ISL_11798568_A/barnacle_goose/Denmark/24273−1.02/2021−10−26_H5N1_PA_2021−10−26
EPI_ISL_9117228_A/northern_goshawk/Netherlands/22000305−002/2022_H5N1_PA_2022−01−04
EPI_ISL_6761007_A/mute_swan/Netherlands/21038362−002/2021_H5N1_PA_2021−11−09
EPI_ISL_6761006_A/mute_swan/Netherlands/21038362−001/2021_H5N1_PA_2021−11−09
EPI_ISL_11561593_A/black−headed_gull/England/388256/2022_H5N1_PA_2022−02−03
EPI_ISL_6328001_A/greylag_goose/Netherlands/21037497−001/2021_H5N1_PA_2021−10−19
EPI_ISL_6761019_A/mute_swan/Netherlands/21038706−002/2021_H5N1_PA_2021−11−14
EPI_ISL_6761000_A/greylag_goose/Netherlands/21037809−001/2021_H5N1_PA_2021−10−31
EPI_ISL_7267245_A/mute_swan/Netherlands/21038797−001/2021_H5N1_PA_2021−11−13
EPI_ISL_7683138_A/fox/Netherlands/21040099−007/2021_H5N1_PA_2021−12−03
EPI_ISL_7683137_A/fox/Netherlands/21040099−006/2021_H5N1_PA_2021−12−03
EPI_ISL_7683118_A/fox/Netherlands/21040099−001/2021_H5N1_PA_2021−12−03
EPI_ISL_9377021_A/goose/France/21P014207/2021_H5N1_PA_2021−12−23
EPI_ISL_6761020_A/mute_swan/Netherlands/21038479−002/2021_H5N1_PA_2021−11−09
EPI_ISL_9261749_A/Caspian_gull/Netherlands/1/2022_H5N1_PA_2022−01−14
EPI_ISL_11019784_A/red_fox/Netherlands/22000097−005/2022_H5N1_PA_2022−01−03
EPI_ISL_11019783_A/red_fox/Netherlands/22000097−001/2022_H5N1_PA_2022−01−03
EPI_ISL_8650947_A/red_fox/Netherlands/22000097−004/2022_H5N1_PA_2022−01−03
EPI_ISL_7778880_A/Red_fox/Estonia/TA2126820_21VIR10433−13/2021_H5N1_PA_2021−11−08
EPI_ISL_7952125_A/mute_swan/Netherlands/21039824−002/2021_H5N1_PA_2021−11−25
EPI_ISL_7054529_A/greylag_goose_/Sweden/SVA211111SZ0376/FB004497/M−2021_H5N1_PA_2021−11−08
EPI_ISL_7635907_A/white−tailed_eagle/Sweden/SVA211201SZ0380/FB004721/M−2021_H5N1_PA_2021−11−26
EPI_ISL_7049600_A/bean_goose/Sweden/SVA211111SZ0372/FB004482/2021_H5N1_PA_2021−11−05
EPI_ISL_2227278_A/goose/Netherlands/21028502−002/21028502/2021_H5N1_PA_2021−05−09
EPI_ISL_2194014_A/white−tailed_eagle/Netherlands/21027616−001/2021_H5N1_PA_2021−04−23
EPI_ISL_2194219_A/red_fox/Netherlands/21028774−004/2021_H5N1_PA_2021−05−17
EPI_ISL_2194218_A/red_fox/Netherlands/21028774−002/2021_H5N1_PA_2021−05−17
EPI_ISL_2227276_A/barnacle_goose/Netherlands/21027357−002/2021_H5N1_PA_2021−04−20
EPI_ISL_7267244_A/common_buzzard/Netherlands/21038793−001/2021_H5N1_PA_2021−11−12
EPI_ISL_6675755_A/goose/Netherlands/21038413−002/2021_H5N1_PA_2021−11−08
EPI_ISL_5804788_A/mute_swan/Netherlands/21037283−002/2021_H5N1_PA_2021−10−24
EPI_ISL_7952129_A/western_jackdaw/Netherlands/21039297−002/2021_H5N1_PA_2021−11−20
Root
0.002

HA

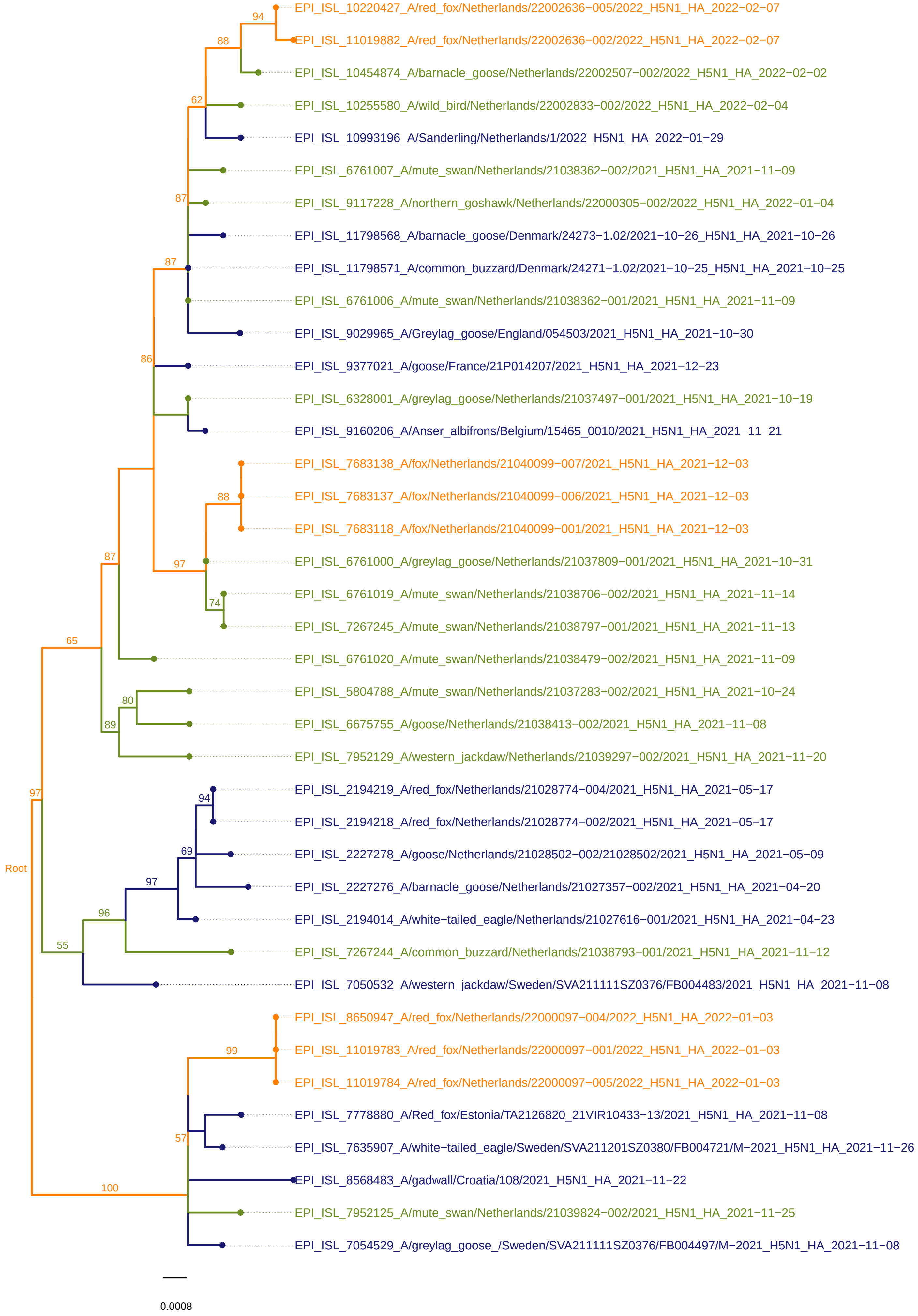
EPI_ISL_10220427_A/red_fox/Netherlands/22002636−005/2022_H5N1_HA_2022−02−07
EPI_ISL_11019882_A/red_fox/Netherlands/22002636−002/2022_H5N1_HA_2022−02−07
EPI_ISL_10454874_A/barnacle_goose/Netherlands/22002507−002/2022_H5N1_HA_2022−02−02
EPI_ISL_10255580_A/wild_bird/Netherlands/22002833−002/2022_H5N1_HA_2022−02−04
EPI_ISL_10993196_A/Sanderling/Netherlands/1/2022_H5N1_HA_2022−01−29
EPI_ISL_6761007_A/mute_swan/Netherlands/21038362−002/2021_H5N1_HA_2021−11−09
EPI_ISL_9117228_A/northern_goshawk/Netherlands/22000305−002/2022_H5N1_HA_2022−01−04
EPI_ISL_11798568_A/barnacle_goose/Denmark/24273−1.02/2021−10−26_H5N1_HA_2021−10−26
EPI_ISL_11798571_A/common_buzzard/Denmark/24271−1.02/2021−10−25_H5N1_HA_2021−10−25
EPI_ISL_6761006_A/mute_swan/Netherlands/21038362−001/2021_H5N1_HA_2021−11−09
EPI_ISL_9029965_A/Greylag_goose/England/054503/2021_H5N1_HA_2021−10−30
EPI_ISL_9377021_A/goose/France/21P014207/2021_H5N1_HA_2021−12−23
EPI_ISL_6328001_A/greylag_goose/Netherlands/21037497−001/2021_H5N1_HA_2021−10−19
EPI_ISL_9160206_A/Anser_albifrons/Belgium/15465_0010/2021_H5N1_HA_2021−11−21
EPI_ISL_7683138_A/fox/Netherlands/21040099−007/2021_H5N1_HA_2021−12−03
EPI_ISL_7683137_A/fox/Netherlands/21040099−006/2021_H5N1_HA_2021−12−03
EPI_ISL_7683118_A/fox/Netherlands/21040099−001/2021_H5N1_HA_2021−12−03
EPI_ISL_6761000_A/greylag_goose/Netherlands/21037809−001/2021_H5N1_HA_2021−10−31
EPI_ISL_6761019_A/mute_swan/Netherlands/21038706−002/2021_H5N1_HA_2021−11−14
EPI_ISL_7267245_A/mute_swan/Netherlands/21038797−001/2021_H5N1_HA_2021−11−13
EPI_ISL_6761020_A/mute_swan/Netherlands/21038479−002/2021_H5N1_HA_2021−11−09
EPI_ISL_5804788_A/mute_swan/Netherlands/21037283−002/2021_H5N1_HA_2021−10−24
EPI_ISL_6675755_A/goose/Netherlands/21038413−002/2021_H5N1_HA_2021−11−08
EPI_ISL_7952129_A/western_jackdaw/Netherlands/21039297−002/2021_H5N1_HA_2021−11−20
EPI_ISL_2194219_A/red_fox/Netherlands/21028774−004/2021_H5N1_HA_2021−05−17
EPI_ISL_2194218_A/red_fox/Netherlands/21028774−002/2021_H5N1_HA_2021−05−17
EPI_ISL_2227278_A/goose/Netherlands/21028502−002/21028502/2021_H5N1_HA_2021−05−09
EPI_ISL_2227276_A/barnacle_goose/Netherlands/21027357−002/2021_H5N1_HA_2021−04−20
EPI_ISL_2194014_A/white−tailed_eagle/Netherlands/21027616−001/2021_H5N1_HA_2021−04−23
EPI_ISL_7267244_A/common_buzzard/Netherlands/21038793−001/2021_H5N1_HA_2021−11−12
EPI_ISL_7050532_A/western_jackdaw/Sweden/SVA211111SZ0376/FB004483/2021_H5N1_HA_2021−11−08
EPI_ISL_8650947_A/red_fox/Netherlands/22000097−004/2022_H5N1_HA_2022−01−03
EPI_ISL_11019783_A/red_fox/Netherlands/22000097−001/2022_H5N1_HA_2022−01−03
EPI_ISL_11019784_A/red_fox/Netherlands/22000097−005/2022_H5N1_HA_2022−01−03
EPI_ISL_7778880_A/Red_fox/Estonia/TA2126820_21VIR10433−13/2021_H5N1_HA_2021−11−08
EPI_ISL_7635907_A/white−tailed_eagle/Sweden/SVA211201SZ0380/FB004721/M−2021_H5N1_HA_2021−11−26
EPI_ISL_8568483_A/gadwall/Croatia/108/2021_H5N1_HA_2021−11−22
EPI_ISL_7952125_A/mute_swan/Netherlands/21039824−002/2021_H5N1_HA_2021−11−25
EPI_ISL_7054529_A/greylag_goose_/Sweden/SVA211111SZ0376/FB004497/M−2021_H5N1_HA_2021−11−08
Root
0.0008

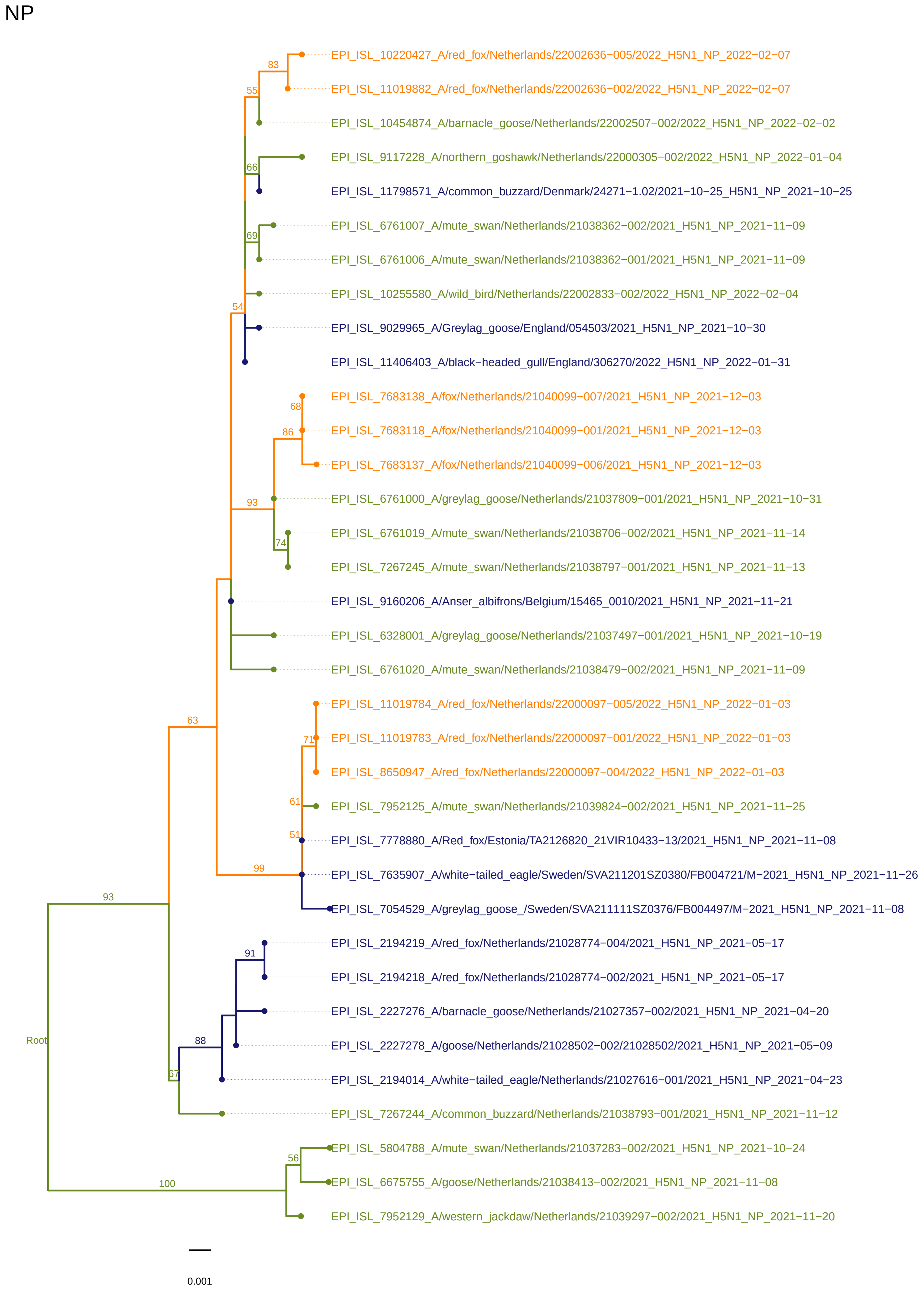
NP
EPI_ISL_10220427_A/red_fox/Netherlands/22002636−005/2022_H5N1_NP_2022−02−07
EPI_ISL_11019882_A/red_fox/Netherlands/22002636−002/2022_H5N1_NP_2022−02−07
EPI_ISL_10454874_A/barnacle_goose/Netherlands/22002507−002/2022_H5N1_NP_2022−02−02
EPI_ISL_9117228_A/northern_goshawk/Netherlands/22000305−002/2022_H5N1_NP_2022−01−04
EPI_ISL_11798571_A/common_buzzard/Denmark/24271−1.02/2021−10−25_H5N1_NP_2021−10−25
EPI_ISL_6761007_A/mute_swan/Netherlands/21038362−002/2021_H5N1_NP_2021−11−09
EPI_ISL_6761006_A/mute_swan/Netherlands/21038362−001/2021_H5N1_NP_2021−11−09
EPI_ISL_10255580_A/wild_bird/Netherlands/22002833−002/2022_H5N1_NP_2022−02−04
EPI_ISL_9029965_A/Greylag_goose/England/054503/2021_H5N1_NP_2021−10−30
EPI_ISL_11406403_A/black−headed_gull/England/306270/2022_H5N1_NP_2022−01−31
EPI_ISL_7683138_A/fox/Netherlands/21040099−007/2021_H5N1_NP_2021−12−03
EPI_ISL_7683118_A/fox/Netherlands/21040099−001/2021_H5N1_NP_2021−12−03
EPI_ISL_7683137_A/fox/Netherlands/21040099−006/2021_H5N1_NP_2021−12−03
EPI_ISL_6761000_A/greylag_goose/Netherlands/21037809−001/2021_H5N1_NP_2021−10−31
EPI_ISL_6761019_A/mute_swan/Netherlands/21038706−002/2021_H5N1_NP_2021−11−14
EPI_ISL_7267245_A/mute_swan/Netherlands/21038797−001/2021_H5N1_NP_2021−11−13
EPI_ISL_9160206_A/Anser_albifrons/Belgium/15465_0010/2021_H5N1_NP_2021−11−21
EPI_ISL_6328001_A/greylag_goose/Netherlands/21037497−001/2021_H5N1_NP_2021−10−19
EPI_ISL_6761020_A/mute_swan/Netherlands/21038479−002/2021_H5N1_NP_2021−11−09
EPI_ISL_11019784_A/red_fox/Netherlands/22000097−005/2022_H5N1_NP_2022−01−03
EPI_ISL_11019783_A/red_fox/Netherlands/22000097−001/2022_H5N1_NP_2022−01−03
EPI_ISL_8650947_A/red_fox/Netherlands/22000097−004/2022_H5N1_NP_2022−01−03
EPI_ISL_7952125_A/mute_swan/Netherlands/21039824−002/2021_H5N1_NP_2021−11−25
EPI_ISL_7778880_A/Red_fox/Estonia/TA2126820_21VIR10433−13/2021_H5N1_NP_2021−11−08
EPI_ISL_7635907_A/white−tailed_eagle/Sweden/SVA211201SZ0380/FB004721/M−2021_H5N1_NP_2021−11−26
EPI_ISL_7054529_A/greylag_goose_/Sweden/SVA211111SZ0376/FB004497/M−2021_H5N1_NP_2021−11−08
EPI_ISL_2194219_A/red_fox/Netherlands/21028774−004/2021_H5N1_NP_2021−05−17
EPI_ISL_2194218_A/red_fox/Netherlands/21028774−002/2021_H5N1_NP_2021−05−17
EPI_ISL_2227276_A/barnacle_goose/Netherlands/21027357−002/2021_H5N1_NP_2021−04−20
EPI_ISL_2227278_A/goose/Netherlands/21028502−002/21028502/2021_H5N1_NP_2021−05−09
EPI_ISL_2194014_A/white−tailed_eagle/Netherlands/21027616−001/2021_H5N1_NP_2021−04−23
EPI_ISL_7267244_A/common_buzzard/Netherlands/21038793−001/2021_H5N1_NP_2021−11−12
EPI_ISL_5804788_A/mute_swan/Netherlands/21037283−002/2021_H5N1_NP_2021−10−24
EPI_ISL_6675755_A/goose/Netherlands/21038413−002/2021_H5N1_NP_2021−11−08
EPI_ISL_7952129_A/western_jackdaw/Netherlands/21039297−002/2021_H5N1_NP_2021−11−20
83
55
66
69
54
68
86
93
74
63
71
61
51
99
93
91
88
67
100
56
Root
0.001

NA

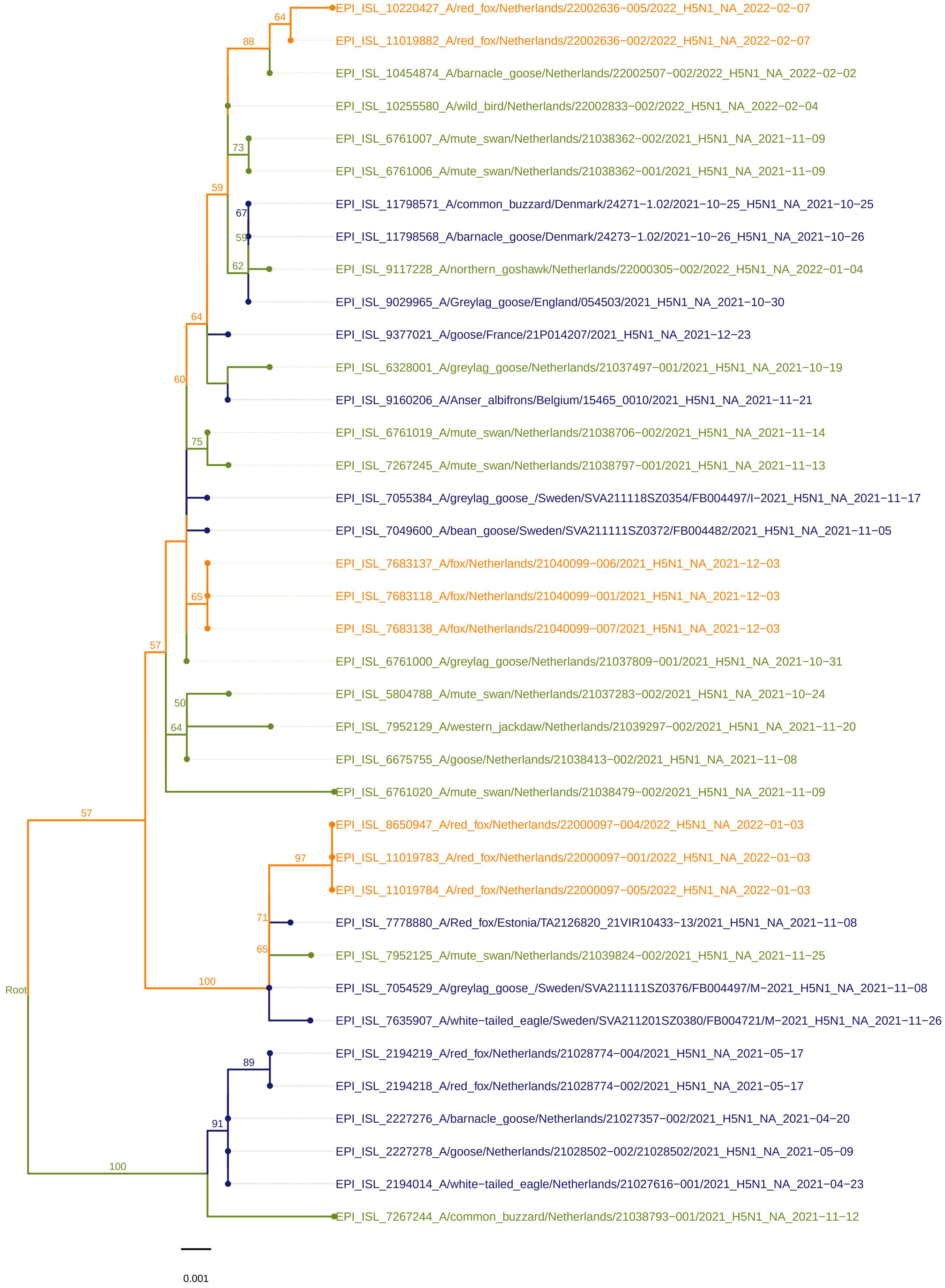

EPI_ISL_10220427_A/red_fox/Netherlands/22002636−005/2022_H5N1_NA_2022−02−07
EPI_ISL_11019882_A/red_fox/Netherlands/22002636−002/2022_H5N1_NA_2022−02−07
EPI_ISL_10454874_A/barnacle_goose/Netherlands/22002507−002/2022_H5N1_NA_2022−02−02
EPI_ISL_10255580_A/wild_bird/Netherlands/22002833−002/2022_H5N1_NA_2022−02−04
EPI_ISL_6761007_A/mute_swan/Netherlands/21038362−002/2021_H5N1_NA_2021−11−09
EPI_ISL_6761006_A/mute_swan/Netherlands/21038362−001/2021_H5N1_NA_2021−11−09
EPI_ISL_11798571_A/common_buzzard/Denmark/24271−1.02/2021−10−25_H5N1_NA_2021−10−25
EPI_ISL_11798568_A/barnacle_goose/Denmark/24273−1.02/2021−10−26_H5N1_NA_2021−10−26
EPI_ISL_9117228_A/northern_goshawk/Netherlands/22000305−002/2022_H5N1_NA_2022−01−04
EPI_ISL_9029965_A/Greylag_goose/England/054503/2021_H5N1_NA_2021−10−30
EPI_ISL_9377021_A/goose/France/21P014207/2021_H5N1_NA_2021−12−23
EPI_ISL_6328001_A/greylag_goose/Netherlands/21037497−001/2021_H5N1_NA_2021−10−19
EPI_ISL_9160206_A/Anser_albifrons/Belgium/15465_0010/2021_H5N1_NA_2021−11−21
EPI_ISL_6761019_A/mute_swan/Netherlands/21038706−002/2021_H5N1_NA_2021−11−14
EPI_ISL_7267245_A/mute_swan/Netherlands/21038797−001/2021_H5N1_NA_2021−11−13
EPI_ISL_7055384_A/greylag_goose_/Sweden/SVA211118SZ0354/FB004497/I−2021_H5N1_NA_2021−11−17
EPI_ISL_7049600_A/bean_goose/Sweden/SVA211111SZ0372/FB004482/2021_H5N1_NA_2021−11−05
EPI_ISL_7683137_A/fox/Netherlands/21040099−006/2021_H5N1_NA_2021−12−03
EPI_ISL_7683118_A/fox/Netherlands/21040099−001/2021_H5N1_NA_2021−12−03
EPI_ISL_7683138_A/fox/Netherlands/21040099−007/2021_H5N1_NA_2021−12−03
EPI_ISL_6761000_A/greylag_goose/Netherlands/21037809−001/2021_H5N1_NA_2021−10−31
EPI_ISL_5804788_A/mute_swan/Netherlands/21037283−002/2021_H5N1_NA_2021−10−24
EPI_ISL_7952129_A/western_jackdaw/Netherlands/21039297−002/2021_H5N1_NA_2021−11−20
EPI_ISL_6675755_A/goose/Netherlands/21038413−002/2021_H5N1_NA_2021−11−08
EPI_ISL_6761020_A/mute_swan/Netherlands/21038479−002/2021_H5N1_NA_2021−11−09
EPI_ISL_8650947_A/red_fox/Netherlands/22000097−004/2022_H5N1_NA_2022−01−03
EPI_ISL_11019783_A/red_fox/Netherlands/22000097−001/2022_H5N1_NA_2022−01−03
EPI_ISL_11019784_A/red_fox/Netherlands/22000097−005/2022_H5N1_NA_2022−01−03
EPI_ISL_7778880_A/Red_fox/Estonia/TA2126820_21VIR10433−13/2021_H5N1_NA_2021−11−08
EPI_ISL_7952125_A/mute_swan/Netherlands/21039824−002/2021_H5N1_NA_2021−11−25
EPI_ISL_7054529_A/greylag_goose_/Sweden/SVA211111SZ0376/FB004497/M−2021_H5N1_NA_2021−11−08
EPI_ISL_7635907_A/white−tailed_eagle/Sweden/SVA211201SZ0380/FB004721/M−2021_H5N1_NA_2021−11−26
EPI_ISL_2194219_A/red_fox/Netherlands/21028774−004/2021_H5N1_NA_2021−05−17
EPI_ISL_2194218_A/red_fox/Netherlands/21028774−002/2021_H5N1_NA_2021−05−17
EPI_ISL_2227276_A/barnacle_goose/Netherlands/21027357−002/2021_H5N1_NA_2021−04−20
EPI_ISL_2227278_A/goose/Netherlands/21028502−002/21028502/2021_H5N1_NA_2021−05−09
EPI_ISL_2194014_A/white−tailed_eagle/Netherlands/21027616−001/2021_H5N1_NA_2021−04−23
EPI_ISL_7267244_A/common_buzzard/Netherlands/21038793−001/2021_H5N1_NA_2021−11−12
Root
0.001

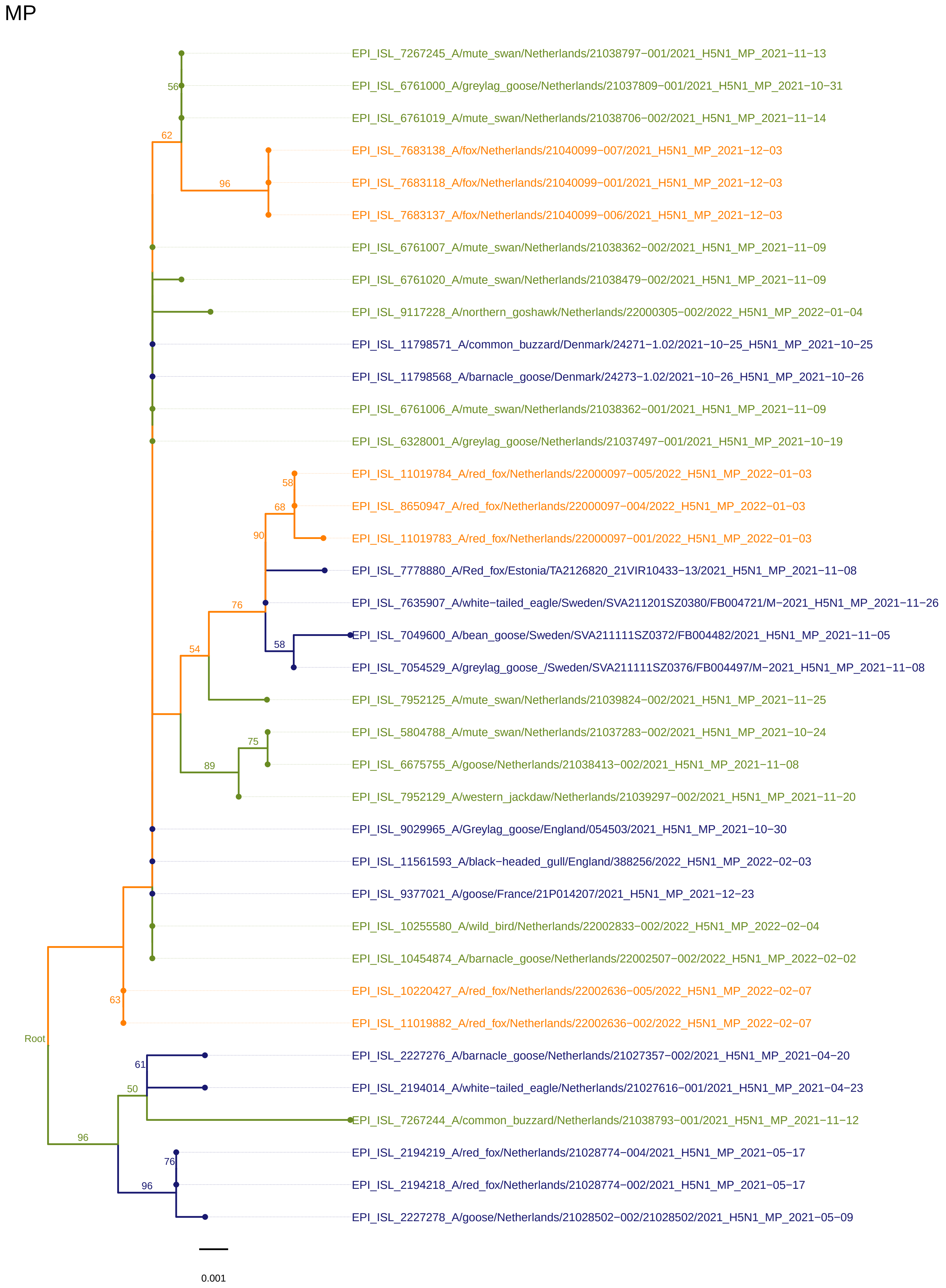
MP
EPI_ISL_7267245_A/mute_swan/Netherlands/21038797−001/2021_H5N1_MP_2021−11−13
EPI_ISL_6761000_A/greylag_goose/Netherlands/21037809−001/2021_H5N1_MP_2021−10−31
EPI_ISL_6761019_A/mute_swan/Netherlands/21038706−002/2021_H5N1_MP_2021−11−14
EPI_ISL_7683138_A/fox/Netherlands/21040099−007/2021_H5N1_MP_2021−12−03
EPI_ISL_7683118_A/fox/Netherlands/21040099−001/2021_H5N1_MP_2021−12−03
EPI_ISL_7683137_A/fox/Netherlands/21040099−006/2021_H5N1_MP_2021−12−03
EPI_ISL_6761007_A/mute_swan/Netherlands/21038362−002/2021_H5N1_MP_2021−11−09
EPI_ISL_6761020_A/mute_swan/Netherlands/21038479−002/2021_H5N1_MP_2021−11−09
EPI_ISL_9117228_A/northern_goshawk/Netherlands/22000305−002/2022_H5N1_MP_2022−01−04
EPI_ISL_11798571_A/common_buzzard/Denmark/24271−1.02/2021−10−25_H5N1_MP_2021−10−25
EPI_ISL_11798568_A/barnacle_goose/Denmark/24273−1.02/2021−10−26_H5N1_MP_2021−10−26
EPI_ISL_6761006_A/mute_swan/Netherlands/21038362−001/2021_H5N1_MP_2021−11−09
EPI_ISL_6328001_A/greylag_goose/Netherlands/21037497−001/2021_H5N1_MP_2021−10−19
EPI_ISL_11019784_A/red_fox/Netherlands/22000097−005/2022_H5N1_MP_2022−01−03
EPI_ISL_8650947_A/red_fox/Netherlands/22000097−004/2022_H5N1_MP_2022−01−03
EPI_ISL_11019783_A/red_fox/Netherlands/22000097−001/2022_H5N1_MP_2022−01−03
EPI_ISL_7778880_A/Red_fox/Estonia/TA2126820_21VIR10433−13/2021_H5N1_MP_2021−11−08
EPI_ISL_7635907_A/white−tailed_eagle/Sweden/SVA211201SZ0380/FB004721/M−2021_H5N1_MP_2021−11−26
EPI_ISL_7049600_A/bean_goose/Sweden/SVA211111SZ0372/FB004482/2021_H5N1_MP_2021−11−05
EPI_ISL_7054529_A/greylag_goose_/Sweden/SVA211111SZ0376/FB004497/M−2021_H5N1_MP_2021−11−08
EPI_ISL_7952125_A/mute_swan/Netherlands/21039824−002/2021_H5N1_MP_2021−11−25
EPI_ISL_5804788_A/mute_swan/Netherlands/21037283−002/2021_H5N1_MP_2021−10−24
EPI_ISL_6675755_A/goose/Netherlands/21038413−002/2021_H5N1_MP_2021−11−08
EPI_ISL_7952129_A/western_jackdaw/Netherlands/21039297−002/2021_H5N1_MP_2021−11−20
EPI_ISL_9029965_A/Greylag_goose/England/054503/2021_H5N1_MP_2021−10−30
EPI_ISL_11561593_A/black−headed_gull/England/388256/2022_H5N1_MP_2022−02−03
EPI_ISL_9377021_A/goose/France/21P014207/2021_H5N1_MP_2021−12−23
EPI_ISL_10255580_A/wild_bird/Netherlands/22002833−002/2022_H5N1_MP_2022−02−04
EPI_ISL_10454874_A/barnacle_goose/Netherlands/22002507−002/2022_H5N1_MP_2022−02−02
EPI_ISL_10220427_A/red_fox/Netherlands/22002636−005/2022_H5N1_MP_2022−02−07
EPI_ISL_11019882_A/red_fox/Netherlands/22002636−002/2022_H5N1_MP_2022−02−07
EPI_ISL_2227276_A/barnacle_goose/Netherlands/21027357−002/2021_H5N1_MP_2021−04−20
EPI_ISL_2194014_A/white−tailed_eagle/Netherlands/21027616−001/2021_H5N1_MP_2021−04−23
EPI_ISL_7267244_A/common_buzzard/Netherlands/21038793−001/2021_H5N1_MP_2021−11−12
EPI_ISL_2194219_A/red_fox/Netherlands/21028774−004/2021_H5N1_MP_2021−05−17
EPI_ISL_2194218_A/red_fox/Netherlands/21028774−002/2021_H5N1_MP_2021−05−17
EPI_ISL_2227278_A/goose/Netherlands/21028502−002/21028502/2021_H5N1_MP_2021−05−09
Root
0.001

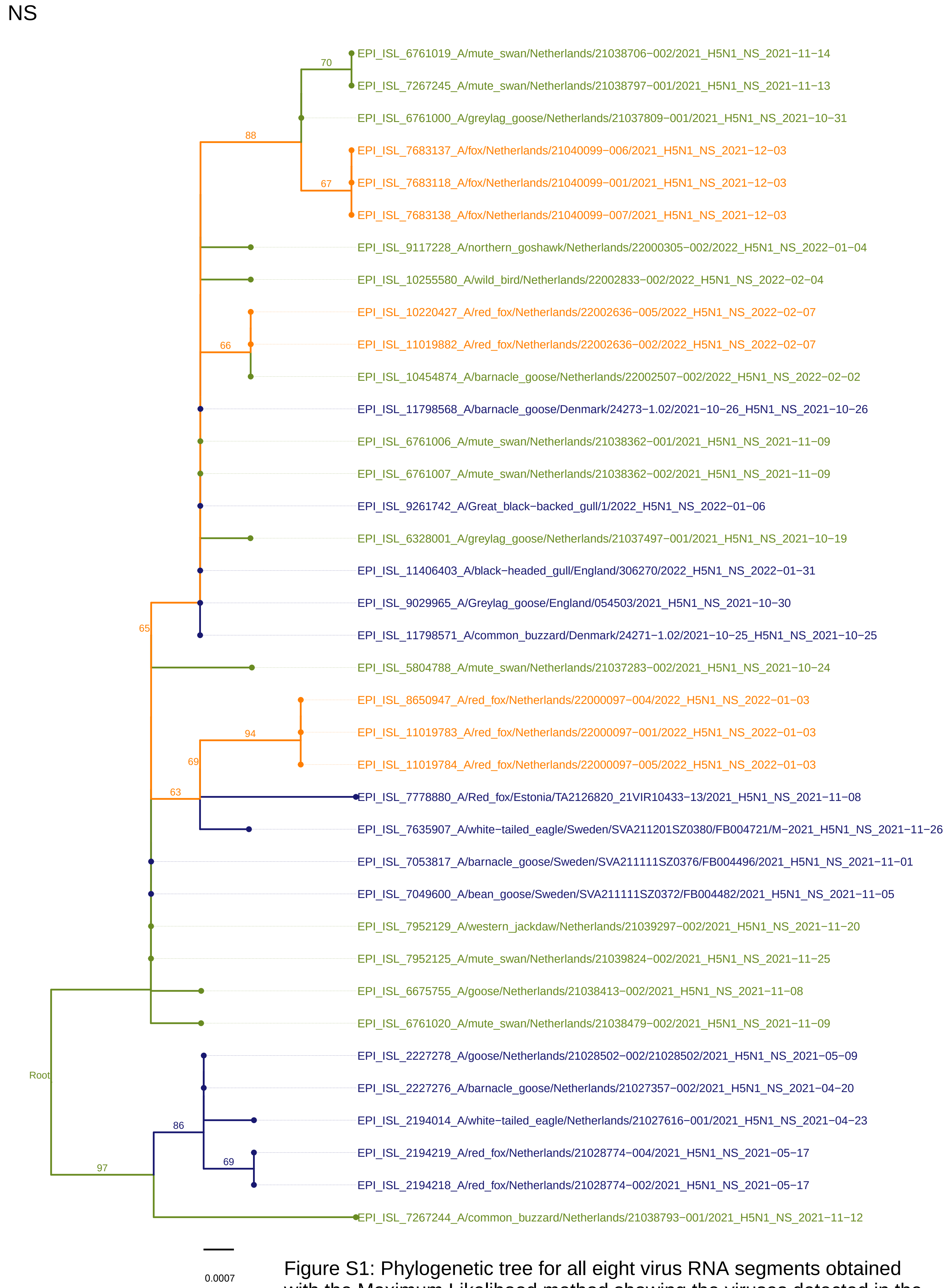

Figure S1: Phylogenetic tree for all eight virus RNA segments obtained with the Maximum Likelihood method showing the viruses detected in the samples of the three foxes (orange), and closely related viral RNA sequences from other viruses detected in the Netherlands (green), Europe (blue) and relevant sequences form the 2020-2021 epizootic

Table S2: Geographical and nucleotide distance between foxes and wild birds infected with HPAI.

Red fox	Sample date	EPI-nr	Sample type	X	Y	Closest wild bird virus	Wild bird species	Sample date	X	Y	Nt differences	Amino acid differences (wild bird to fox throat swab)	Days since detection closest wild bird virus	Distance between locations (km)
**Dorst**	2021-12-03	EPI ISL 7683118	Throat swab	104419	389025	EPI ISL 6761000	greylag goose	2021-10-31	116555	425951	17	**PB2**-Q13H, K332R, A588V **PA**-N115S **HA**-E201K **NP**-A234S **M2**-T65K	-33	38,9
		EPI ISL 7683137	Amnion horn and medulla oblongata											
		EPI ISL 7683138	Cerebrum and cerebellum											
**Heemskerk**	2022-01-03	EPI ISL 11019783	Throat swab	105024	502339	EPI ISL 7952125	mute swan	2021-11-25	208553	591693	42	**PB1-F2** N17S, K73R **PB1**-I108L, I113V, L150F, K531R **PA**-L532I **HA**-R339K **NA**-E77K, S148T **M1**-G96A, T182A **M2**-I28F **NS1**-P215S, K221Q	-39	136,8
		EPI ISL 8650947	Amnion horn and medulla oblongata											
		EPI ISL 11019784	Cerebrum and cerebellum											
**Oosterbeek**	2022-02-07	EPI ISL 11019882	Throat swab	186535	444255	EPI_ISL 10454874	barnacle goose	2022-02-02	208553	591693	22	**PB2**-A278T, Q447R **PB1**-N76D, T182I, G538D **PA**-T20A, F277S **HA**-L7F, K46Q, S288G **NA**-V94I	-5	149,1
		EPI ISL 10220427	Cerebrum and cerebellum											

Table S3: MLL analysis of variables time and virus variants 627E-627K.

Time [h]	Temperature [°C]	Estimate	SE	df	t.ratio	p.value
**12**	33	-0.0903	0.231	5.01	-0.391	0.7120
**24**	33	-0.3993	0.231	5.01	-1.729	0.1443
**48**	33	-0.9167	0.231	5.01	-3.969	0.0106
**72**	33	-0.9792	0.231	5.01	-4.239	0.0081
**12**	37	-0.0903	0.231	5.01	-0.391	0.7120
**24**	37	-0.3993	0.231	5.01	-1.729	0.1443
**48**	37	-0.9167	0.231	5.01	-3.969	0.0106
**72**	37	-0.9792	0.231	5.01	-4.239	0.0081

Table S4: MLL analysis of variables time and temperatures 33°C and 37°C.

Time [h]	PB2 variant	Estimate	SE	df	t.ratio	p.value
**12**	627E	0.00694	0.168	272	0.041	0.9671
**12**	627K	0.00694	0.168	272	0.041	0.9671
**24**	627E	-1.28125	0.168	272	-7.619	<0.0001
**24**	627K	-1.28125	0.168	272	-7.619	<0.0001
**48**	627E	-0.40792	0.168	272	-2.436	0.0155
**48**	627K	-0.40792	0.168	272	-2.436	0.0155
**72**	627E	0.39583	0.168	272	2.354	0.0193
**72**	627K	0.39583	0.168	272	2.354	0.0193

Table S5: GISAID accession numbers

We gratefully acknowledge the authors, originating and submitting laboratories of the sequences from GISAID's EpiFlu™ Database on which this research is based. The list is detailed below.
All submitters of data may be contacted directly via www.gisaid.org

Isolate-ID	Country	Collection date	Isolate name	Originating Lab	Submitting Lab	Authors
EPI_ISL_9377021	France	2021-Dec-23	A/goose/France/21P014207/2021	Anses (Ploufragan-Plouzané)	ANSES Agence Nationale De Securite Sanitaire De L'alimentation	
EPI_ISL_6675755	Netherlands	2021-Nov-08	A/goose/Netherlands/21038413-002/2021	Wageningen Bioveterinary Research	Wageningen Bioveterinary Research	Beerens, Nancy; Harders, Frank; Pritz-Verschuren, Sylvia; Roose, Marit; Germeraad, Evelien; Engelsma, Marc; Heutink, Rene
EPI_ISL_2227278	Netherlands	2021-May-09	A/goose/Netherlands/21028502-002/21028502/2021	Wageningen Bioveterinary Research	Wageningen Bioveterinary Research	Beerens, Nancy; Harders, Frank; Pritz-Verschuren, Sylvia; Roose, Marit; Germeraad, Evelien; Engelsma, Marc; Bossers, Alex; Heutink, Rene
EPI_ISL_9029965	United Kingdo	2021-Oct-30	A/Greylag_goose/England/054503/2021	Animal and Plant Health Agency (APHA)	Animal and Plant Health Agency (APHA)	
EPI_ISL_6761000	Netherlands	2021-Oct-31	A/greylag goose/Netherlands/21037809-001/2021	Wageningen Bioveterinary Research	Wageningen Bioveterinary Research	Beerens, Nancy; Harders, Frank; Pritz-Verschuren, Sylvia; Roose, Marit; Germeraad, Evelien; Engelsma, Marc; Heutink, Rene
EPI_ISL_6328001	Netherlands	2021-Oct-19	A/greylag goose/Netherlands/21037497-001/2021	Wageningen Bioveterinary Research	Wageningen Bioveterinary Research	Beerens, Nancy; Harders, Frank; Pritz-Verschuren, Sylvia; Roose, Marit; Germeraad, Evelien; Engelsma, Marc; Heutink, Rene
EPI_ISL_7952125	Netherlands	2021-Nov-25	A/mute swan/Netherlands/21039824-002/2021	Wageningen Bioveterinary Research	Wageningen Bioveterinary Research	Beerens, Nancy; Harders, Frank; Pritz-Verschuren, Sylvia; Roose, Marit; Venema, Sandra; Germeraad, Evelien; Engelsma, Marc; Heutink, Rene
EPI_ISL_6761020	Netherlands	2021-Nov-09	A/mute swan/Netherlands/21038479-002/2021	Wageningen Bioveterinary Research	Wageningen Bioveterinary Research	Beerens, Nancy; Harders, Frank; Pritz-Verschuren, Sylvia; Roose, Marit; Germeraad, Evelien; Engelsma, Marc; Heutink, Rene
EPI_ISL_5804788	Netherlands	2021-Oct-24	A/Mute swan/Netherlands/21037283-002/2021	Wageningen Bioveterinary Research	Wageningen Bioveterinary Research	Beerens, Nancy; Harders, Frank; Pritz-Verschuren, Sylvia; Roose, Marit; Germeraad, Evelien; Engelsma, Marc; Heutink, Rene
EPI_ISL_7952129	Netherlands	2021-Nov-20	A/western jackdaw/Netherlands/21039297-002/2021	Wageningen Bioveterinary Research	Wageningen Bioveterinary Research	Beerens, Nancy; Harders, Frank; Pritz-Verschuren, Sylvia; Roose, Marit; Venema, Sandra; Germeraad, Evelien; Engelsma, Marc; Heutink, Rene
EPI_ISL_2194219	Netherlands	2021-May-17	A/red fox/Netherlands/21028774-004/2021	Wageningen Bioveterinary Research	Wageningen Bioveterinary Research	Beerens, Nancy; Harders, Frank; Pritz-Verschuren, Sylvia; Roose, Marit; Germeraad, Evelien; Engelsma, Marc; Bossers, Alex; Heutink, Rene
EPI_ISL_7778880	Estonia	2021-Nov-08	A/Red_fox/Estonia/TA2126820_21VIR10433-13/2021	Estonian Veterinary and Food Laboratory	Istituto Zooprofilattico Sperimentale Delle Venezie	Nurmoja, I.; Vilem, A.; Juurik, T.; Zecchin, B.; Fusaro, A.; Schivo, A.; Salviato, A.; Palumbo, E.; Milani, A.; Giussani, E.; Pastori, A.; Monne, I.; Terregino, C.
EPI_ISL_2194218	Netherlands	2021-May-17	A/red fox/Netherlands/21028774-002/2021	Wageningen Bioveterinary Research	Wageningen Bioveterinary Research	Beerens, Nancy; Harders, Frank; Pritz-Verschuren, Sylvia; Roose, Marit; Germeraad, Evelien; Engelsma, Marc; Bossers, Alex; Heutink, Rene
EPI_ISL_9261751	Netherlands	2022-Jan-14	A/Barnacle goose/Netherlands/6/2022	Erasmus Medical Center	Erasmus Medical Center	
EPI_ISL_9261749	Netherlands	2022-Jan-14	A/Caspian gull/Netherlands/1/2022	Erasmus Medical Center	Erasmus Medical Center	
EPI_ISL_9261742	Netherlands	2022-Jan-06	A/Great black-backed gull/1/2022	Erasmus Medical Center	Erasmus Medical Center	
EPI_ISL_10993196	Netherlands	2022-Jan-29	A/Sanderling/Netherlands/1/2022	Erasmus Medical Center	Erasmus Medical Center	
EPI_ISL_7050532	Sweden	2021-Nov-08	A/western jackdaw/Sweden/SVA211111SZ0376/FB004483/2021	National Veterinary Institute, SVA	National Veterinary Institute	
EPI_ISL_502614	Belgium	2018-Apr-09	A/Anas platyrhynchos/Belgium/7976/2018		Import from public-domain	Lambrecht,B.; Steensels,M.; Fusaro,A.; Milani,A.; Pastori,A.; Schivo,A.; Salviato,A.; Zamperin,G.; Monne,I.; Terregino,C.
EPI_ISL_502613	Belgium	2018-Aug-29	A/Anas platyrhynchos/Belgium/7828/2018		Import from public-domain	Lambrecht,B.; Steensels,M.; Fusaro,A.; Milani,A.; Pastori,A.; Schivo,A.; Salviato,A.; Zamperin,G.; Monne,I.; Terregino,C.
EPI_ISL_8568483	Croatia	2021-Nov-22	A/gadwall/Croatia/108/2021	Croatian Veterinary Institute, Poultry Centre	Croatian Veterinary Institute	Savić, Vladimir
EPI_ISL_9117228	Netherlands	2022-Jan-04	A/northern goshawk/Netherlands/22000305-002/2022	Wageningen Bioveterinary Research	Wageningen Bioveterinary Research	Beerens, Nancy; Harders, Frank; Pritz-Verschuren, Sylvia; Roose, Marit; Venema, Sandra; Germeraad, Evelien; Engelsma, Marc; Heutink, Rene
EPI_ISL_11798571	Denmark	2021-Oct-25	A/common_buzzard/Denmark/24271-1.02/2021-10-25	Statens Serumm Institute	Statens Serum Institute	Charlotte Hjulsager, Yuan Liang

EPI_ISL_7267244	Netherlands	2021-Nov-12	A/common buzzard/Netherlands/21038793-001/2021	Wageningen Bioveterinary Research	Wageningen Bioveterinary Research	Beerens, Nancy; Harders, Frank; Pritz-Verschuren, Sylvia; Roose, Marit; Venema, Sandra; Germeraad, Evelien; Engelsma, Marc; Heutink, Rene
EPI_ISL_2194014	Netherlands	2021-Apr-23	A/white-tailed eagle/Netherlands/21027616-001/2021	Wageningen Bioveterinary Research	Wageningen Bioveterinary Research	Beerens, Nancy; Harders, Frank; Pritz-Verschuren, Sylvia; Roose, Marit; Germeraad, Evelien; Engelsma, Marc; Bossers, Alex; Heutink, Rene
EPI_ISL_7055384	Sweden	2021-Nov-17	A/greylag goose /Sweden/SVA211118SZ0354/FB004497/I-2021	National Veterinary Institute, SVA	National Veterinary Institute	
EPI_ISL_7054529	Sweden	2021-Nov-08	A/greylag goose /Sweden/SVA211111SZ0376/FB004497/M-2021	National Veterinary Institute, SVA	National Veterinary Institute	
EPI_ISL_7049600	Sweden	2021-Nov-05	A/bean goose/Sweden/SVA211111SZ0372/FB004482/2021	National Veterinary Institute, SVA	National Veterinary Institute	
EPI_ISL_7635907	Sweden	2021-Nov-26	A/white-tailed eagle/Sweden/SVA211201SZ0380/FB004721/M-2021	National Veterinary Institute, SVA	National Veterinary Institute	
EPI_ISL_11561593	United Kingdo	2022-Feb-03	A/black-headed_gull/England/388256/2022	Animal and Plant Health Agency (APHA)	Animal and Plant Health Agency (APHA)	
EPI_ISL_11406403	United Kingdo	2022-Jan-31	A/black-headed_gull/England/306270/2022	Animal and Plant Health Agency (APHA)	Animal and Plant Health Agency (APHA)	
EPI_ISL_11798568	Denmark	2021-Oct-26	A/barnacle_goose/Denmark/24273-1.02/2021-10-26	Statens Serumm Institute	Statens Serum Institute	Charlotte Hjulsager, Yuan Liang
EPI_ISL_267243	Netherlands	2014-Dec-15	A/barnacle goose/Netherlands/2/2014		Import from public-domain	
EPI_ISL_2227276	Netherlands	2021-Apr-20	A/barnacle goose/Netherlands/21027357-002/2021	Wageningen Bioveterinary Research	Wageningen Bioveterinary Research	Beerens, Nancy; Harders, Frank; Pritz-Verschuren, Sylvia; Roose, Marit; Germeraad, Evelien; Engelsma, Marc; Bossers, Alex; Heutink, Rene
EPI_ISL_7053817	Sweden	2021-Nov-01	A/barnacle goose/Sweden/SVA211111SZ0376/FB004496/2021	National Veterinary Institute, SVA	National Veterinary Institute	
EPI_ISL_9160206	Belgium	2021-Nov-21	A/Anser_albifrons/Belgium/15465_0010/2021	Sciensano - Animal Infectious Diseases	Sciensano, Department of Animal Infectious Diseases	Van Borm, Steven; Roupie, Virginie; Lambrecht, Benedicte; Mathijs, Elisabeth; Steensels, Mieke
EPI_ISL_403716	Russian Fede	2019-Sep-29	A/Common_Teal/Dagestan/34d/2019	Research Institute of Experimental and Clinical Medicine	National Institute of Animal Health	Mine, J.; Uchida, Y.; Saito, T.; Shestopalov, A.; Dubovitskiy, N.; Sobolev, I.; Derko, A.; Alekseev, A.; Murashkina, T.; Sharshov, K.

Table S6: Primer sequences with Illumina tag for amplification of PB2 protein region.

**AI-PB2-forward**	TCGTCGGCAGCGTCAGATGTGTATAAGAGACAGGGNCAGATGCGTGATGTGCTGG
**AI-PB2-reverse**	GTCTCGTGGGCTCGGAGATGTGTATAAGAGACAGCGGTTGAACACAGGGGAGTTGC
